# Study on the participation of nursing staff in tobacco cessation support and related influencing factors: A survey from Chongqing, China

**DOI:** 10.18332/tid/170753

**Published:** 2023-10-12

**Authors:** Jun Song, Yanhan Chen, Zhiyong Zhang, Yang Cao, Li Zhang

**Affiliations:** 1 Department of Ophthalmology, Chongqing General Hospital, Chongqing, People’s Republic of China; 2 College of Nursing, Chongqing Medical University, Chongqing, People’s Republic of China; 3 Integrated Traditional Chinese Medicine and Western Medicine Department, Healthcare Center, Jinlong Town, People’s Republic of China; 4 The First People's Hospital of Chongqing High-tech Zone, Chongqing, People’s Republic of China

**Keywords:** 5As, nursing intervention, smoking cessation, attitudes about smoking cessation, tobacco control resources

## Abstract

**INTRODUCTION:**

Nursing staff’s assistance for smokers to quit smoking can increase the rate of quitting. The smoking cessation help can be affected by many factors. This study surveyed the use of the 5As (Asking, Advising, Assessing, Assisting, Arranging) approach to support smoking cessation by the nursing staff in Chongqing, China, and analyzed the corresponding influencing factors.

**METHODS:**

A stratified random cluster sampling method was used to select nursing staff from 8 tertiary hospitals, 5 secondary hospitals, 12 community health centers, and 35 township health centers in different geographical regions of Chongqing. A questionnaire survey was conducted among the nursing staff to investigate their participation in smoking cessation. Binary logistic regression analysis was employed to analyze the influencing factors of smoking cessation 5As behavior of the nursing staff.

**RESULTS:**

The 1669 participants were 44 males (2.6%) and 1625 females (97.4%), with an average age of 37.00 ± 10.89 years. Among the participants, 55.2% were from tertiary hospitals, 23.2% from secondary hospitals, 14.2% from township health centers, and 7.4 from community health centers. The often or always used behaviors were: Asking, 69.2%; Advising, 53.0%; Assessing, 39.5%; Assisting, 33.7%; and Arranging, 25.1%. The factors that affected all the 5As were: smoking cessation training (AOR=1.60; 95% CI: 1.22–2.11), knowledge of smoking cessation guidelines (AOR=1.75; 95% CI: 1.32–2.32) and the use of smoking cessation Apps (AOR=1.50; 95% CI: 1.09–2.06), and smokers’ willingness to quit (AOR=2.20; 95% CI: 1.60–3.02).

**CONCLUSIONS:**

Smoker’s motivation to quit smoking and nurses’ knowledge of tobacco cessation resources affected nurses’ participation in smoking control behavior. While encouraging smokers to quit smoking, clinical nursing staff should be provided with related resources to advocate smoking cessation. Guided by the Chinese Clinical Tobacco Cessation Guidelines, smoking control knowledge and skills training should be provided for nursing staff to increase their positive attitude towards smoking control, so as to promote their active participation.

## INTRODUCTION

The tobacco epidemic, one of the most serious threats to human health, causes an annual mortality of more than 8 million worldwide^1^. China is the largest cigarette producer and consumer, with 308 million smokers and over 1 million people dying of tobacco-related diseases each year^2^. In order to curb the tobacco epidemic, China joined the World Health Organization Framework Convention on Tobacco in 2003, which came into force in 2006. Since then, China has drafted a series of tobacco cessation regulations, including banning tobacco advertising, publicizing the dangers of tobacco use through media, prohibiting smoking in public places, establishing smoke-free schools and hospitals, requiring prominent cadres to be the model of quitting smoking, and encouraging medical staff to help smokers to quit^3^. A smoking cessation intervention of 1–3 minutes from medical staff to smokers was found to be effective^4^. Chinese Clinical Smoking Cessation Guidelines^5^ require that all medical personnel perform 5As to help smokers to quit smoking, namely: 1) Asking clients about their smoking status, 2) Advising smokers to quit, 3) Assessing smokers’ willingness to quit, 4) Assisting those who are willing to quit; and 5) Arranging follow-up services for smokers who commit to quitting smoking in order to help remove the challenges they may encounter in the process.

As health specialists and promoters who stay with patients the most time, nurses are best suited for health education and smoking cessation interventions^6^. Studies reveled that the smoking cessation rate increased significantly after nurses provided brief smoking control counseling^7,8^. Currently, the number of nursing staff in China exceeds 5 million, making it the largest healthcare professional personnel^9^. The smoking rate among Chinese nursing staff is extremely low, ranging from 0.7% to 1.8%, making nurses role models for non-smoking^10-12^ and an abundant human resource for smoking control. However, the participation of nursing staff in smoking control is not high, with only about 52% assessing and assisting smokers to quit smoking^11^. Fully understanding the factors that affect nurses’ participation in smoking control intervention could help promote nursing education and develop plans, and then adopt tailored strategies to encourage nurses to participate in smoking control, and help more smokers quit smoking^13^.

Previous studies on nursing staff participation in tobacco cessation analyzed smoking control behaviors and influencing factors in different regions with different cultural background^13^, as well as reports in China. The reported influencing factors included: 1) socioeconomic factors, 2) smoker nurses, 3) smokers’ motivation to quit; and 4) other contributing factors and obstacles^13^. These studies focused on assessing nursing staff competence, lack of training, available time, and knowledge of tobacco cessation^14^. In terms of promoting and hindering factors, the major analysis focused on nurses’ smoking control competence, lack of training, time, and knowledge. Little analyzed has been the knowledge of available smoking cessation resources, such as smoking cessation drugs, applications about smoking cessation, smoking cessation phone lines, and smoking cessation websites, and its impact on smoking control participation of nursing staff.

In addition, studies on Chinese nurses’ involvement in tobacco cessation support have been conducted for nearly 10 years, mainly in tertiary general hospitals which have rich medical resources for smoking cessation^15^. In the last 10 years, smoking cessation regulations and interventions in China have come a long way. The 2030 Goal of Healthy China regulation aims to reduce the smoking rate of adults over 18 years from 27.7% in 2015 to 20% in 2030^16^. In order to achieve this goal, regional tobacco cessation action plans have been developed. Chongqing issued the Smoking Ban in Public Places on 1 January 2021, which was expected to increase both the smokers’ willingness to quit smoking and the demand for smoking cessation services. Nevertheless, some studies found that the public had little knowledge of tobacco cessation resources^17^. Lack of access to smoking cessation resources would affect the willingness to quit smoking and the likelihood of successful smoking cessation. Another study on smoking control among undergraduate nursing students in Chongqing found that students saw fewer of the 5As among clinical nursing staff, and this directly decreased students’ participation in smoking control behaviors^18^. Currently, no study has conducted an extensive smoking control survey among nursing staff in different levels of medical institutions in Chongqing, so the involvement of nurses in tobacco cessation in Chongqing is unclear. Therefore, this study conducted a smoking control survey among nursing staff in Chongqing. Its aim was to investigate the 5As behavior of nursing staff in helping smokers, and to identify the corresponding factors that influence their participation in smoking control.

## METHODS

### Study design

From November to December 2022, stratified random cluster sampling was used to select samples according to the proportion of nursing staff at different levels of medical institutions in Chongqing. The sample size was calculated by the formula for a cross-sectional study. Sarna et al.^11^ found that 52% of nursing staff assessed smokers’ willingness of quitting smoking and assisted smokers to quit. Consequently, we set α=0.05, δ=3%, and a non-response rate of 10%, requiring a total of 1173 nursing staff to be recruited.

Firstly, one administrative district was randomly selected from each of the five regions in the east, west, north, south, and middle of Chongqing. Then, 1–2 hospitals and more than 2 community or township health centers were randomly selected in each of the selected administrative districts. The survey involved 8 tertiary hospitals, 5 secondary hospitals, 12 community health centers, and 35 township health centers. Conversations with the leaders of nursing departments of the selected institutions were conducted to explain the purpose and methods of the survey. After consent was obtained, nursing managers released online questionnaires to their nurses. Participants’ inclusion criterion was: being on-the-job registered nurses providing clinical or community care. Non-nursing staff not directly delivering service to patients were not invited to participate in the study.

### Measures and questionnaire

The questionnaire was developed by the research group. The research group consisted of experts in clinical smoking control, epidemiology, clinical nursing, statistics, and nursing education. Based on the Knowledge-Attitude-Belief-Practice Model, the questionnaire was designed after referring to the Chinese Clinical Smoking Cessation Guidelines^5^, Report on the Health Hazards of Smoking in China 2020^19^, and relevant literature^10-14,20^. After several revisions, it was used to take a pilot survey among 92 nursing staff, to assess the internal consistency of the questionnaire. The pilot survey revealed a Cronbach α=0.942, allowing a feasible formal investigation. The questionnaire included participants’ basic information, knowledge, and attitude towards the 5As approach to helping smokers quit. An informed consent form was attached to the questionnaire with information on the objectives, procedures, potential risks and benefits of participating in the survey. Only after signing the informed consent could participants proceed to the anonymous online survey.

### Sociodemographic factors

Information was obtained on the basic characteristics of participants and included gender, age, professional title, education level, working unit, institution level (tertiary hospital, secondary hospital, community health center, township health center), training in smoking cessation skills, smoking status, exposure to secondhand smoking, and smoking-related diseases of family members and friends.

### Knowledge of tobacco cessation

The survey contained 16 questions/statements in total, 10 on the harmful effects of smoking and secondhand smoking, such as ‘smoking increases the incidence of diabetes ulcers’, and six on the benefits of quitting such as ‘1 year after quitting smoking, the risk of coronary heart disease is half that of smokers’. The response options were: ‘agree’, ‘disagree’, and ‘don’t known’. If the answer was correct, 1 point was given, and zero for an incorrect or ‘don’t know’ response. The higher the score, the more knowledge the participant had about smoking cessation.

### Tobacco cessation resources

The knowledge of tobacco cessation resources was assessed by 5 items, including knowledge of the Chinese Clinical Smoking Cessation Guidelines, smoking cessation Apps, smoking control drugs, smoking cessation phone lines, and smoking cessation websites, with a score from 1 (completely unknown) to 5 (very familiar). The internal consistency had a Cronbach alpha of 0.910.

### Attitude towards tobacco cessation

The attitude towards tobacco cessation was assessed through 9 questions/statements, for example, ‘Nurses are role models of non-smokers. It is the responsibility of nursing staff to help clients to quit smoking’. The answers were scored from 1 (strongly oppose) to 5 (strongly agree), and the internal consistency had a Cronbach alpha of 0.937.

### External environmental factors

External environmental factors affecting nurses’ participation in tobacco cessation support were surveyed by 3 items, including smokers’ motivation to quit, available time, and the working unit’s support. The options were scored from 1 (very unimportant) to 5 (very important). The internal consistency had a Cronbach alpha of 0.773.

### Smoking cessation practice

Five items were used to assess the application of the 5As by nursing staff in the previous year. Each item was scored on a 5-point Likert scale from 1 (never) to 5 (always), and the internal consistency had a Cronbach alpha of 0.865.

### Quality control

The questionnaire was developed with reference to documents, and was revised and pilot tested repeatedly until reliability and validity were ensured. To prevent repeated submission of the questionnaire, the survey was conducted online with a limit of one submission per IP address. Some retrieved questionnaires were deleted for contradictory logic or when the time to fill in the questionnaire was less than 150 seconds.

### Data analysis

SPSS 20.0 software (IBM Corporation, Armonk, NY, US) was used to analyze the data. Descriptive analysis of participants’ characteristics is presented in terms of frequencies and percentages or means and standard deviation. The smoking control support behavior of nursing staff was related to each of the 5As nurses performed when helping smokers quit smoking and the relevant factors involved in each of the 5As. The results were converted from the 5-point Likert scale to binary conversions, scoring 0 (never, rarely, and sometimes) or 1 (often, and always). To identify the factors that promote nursing staff participation in 5As implementation of nursing staff, the 5As behaviors of nursing staff and other variables were analyzed by univariable analysis, including: 1) demographic characteristics and training in smoking cessation, 2) smoking exposure, such as being a smoker nurse or exposed to secondhand smoking, 3) knowledge of and attitude towards smoking cessation, 4) knowledge of tobacco cessation resources; and 5) other external environmental factors. For the knowledge of smoking cessation, correct answers were scored with 1 and incorrect answers with 0, and participants divided into higher and lower average score groups. In terms of attitude, the total score was summed up and participants divided into higher (active) and lower (inactive) average score groups. The variables with statistical significance in univariate analysis (p<0.05) were used for binary logistic regression analysis by the backward method. Statistical significance was set at p<0.05.

## RESULTS

A total of 1780 questionnaires were retrieved, but 111 were excluded because of insufficient filling in time or logical contradictions. Thus, 1669 valid questionnaires remained, with an effective response rate of 93.8%. In the previous year, 1155 (69.2%) of nursing staff often or always asked clients about their smoking status; 885 (53.0%) often or always advised smokers to quit, 660 (39.5%) often or always assessed smoking cessation; 561 (33.7%) often or always assisted clients to quit smoking; 419 (25.1%) often or always arranged follow-up services for smokers’ cessation, and 120 (7.2%) nursing staff always performed all the 5As.

### Univariable analysis of the nurses’ use of the 5As model in tobacco cessation support

*Basic characteristics of nursing staff and their 5As in tobacco cessation*

Univariable analysis was conducted on the nurses’ use of the 5As to support tobacco cessation. Table 1 shows the demographic characteristics and the statistical analysis of nurses’ use of 5As in smoking cessation support. Most nursing staff (97.4%) were female. The percentage of female nurses assessing the smoking status of clients was higher than that of male nurses, with a statistically significant difference (p<0.05). The average age of the participants was 37.00 ± 10.89 years (range: 20–57). The older performed more asking and helping behaviors than the younger did (p<0.05). The number of nurses from the internal medicine department was the largest (29.1%). The behaviors of asking, advising, and assessing differed in different departments, and the difference was statistically significant (p<0.05). Most participants (72.4%) were married. They conducted more asking, advising, assessing and helping than the divorced or unmarried, and the difference was statistically significant (p<0.05). A total of 62.0% of the participants had primary professional titles. Nursing staff with senior professional titles delivered more interventions of advising than those with lower professional titles (p<0.05); 72.0% nursing staff had a Bachelor’s degree or higher (p<0.05). They performed more 5As than those with lower education level (p<0.05) and 55.2% nursing staff were from tertiary A hospitals. Nurses from secondary hospitals and tertiary A hospitals asked more about the clients’ smoking status than those from township healthcare centers or community healthcare centers (p<0.05). Less than half (41.4%) of nurses attended smoking cessation training that was relevant to the 5As behavior change model (p<0.05).

**Table 1:** The relationship between demographic characteristics of nurses and their 5As smoking control behaviors: A survey in 2022 from Chongqing, China (N=1669)

*Variables*	*Total*	*Asking*		*Advising*		*Assessing*		*Assisting*		*Arranging*	
	*n (%)*	*n (%)*	*p*	*n (%)*	*p*	*n (%)*	*p*	*n (%)*	*p*	*n (%)*	*p*
**Gender**			0.002		0.103		0.021		0.220		0.712
Male	44 (2.6)	21 (47.7)		18 (40.9)		10 (22.7)		11 (25.0)		10 (22.7)	
Female	1625 (97.4)	1134 (69.8)		867 (53.4)		650 (40.0)		550 (33.8)		409 (25.2)	
**Age** (years)			0.002		0.065		0.959		0.017		0.529
20–29	661 (39.6)	443 (67.0)		343 (51.9)		256 (38.7)		200 (30.3)		169 (25.6)	
30–39	749 (44.9)	507 (67.7)		386 (51.5)		300 (40.1)		256 (34.2)		192 (25.6)	
40–49	217 (13.0)	171 (78.8)		128 (59.0)		87 (40.1)		85 (39.2)		46 (21.2)	
≥50	42 (2.5)	34 (81.0)		28 (66.7)		17 (40.5)		20 (47.6)		12 (28.6)	
**Marital status**			0.003		0.039		0.013		0.005		0.156
Married	1208 (72.4)	864 (71.5)		662 (54.8)		504 (41.7)		434 (35.9)		316 (26.2)	
Unmarried	404 (24.2)	258 (63.9)		192 (47.5)		137 (33.9)		110 (27.2)		87 (21.5)	
Divorced	57 (3.4)	33 (57.9)		31 (54.4)		19 (33.3)		17 (29.8)		16 (28.1)	
**Education level**			0.079		0.317		0.369		0.046		0.951
Junior and lower	468 (28.0)	309 (66.0)		239 (51.1)		177 (37.8)		140 (29.9)		117 (25.0)	
Undergraduate and higher	1201 (72.0)	846 (70.4)		646 (53.8)		483 (40.2)		421 (35.1)		302 (25.1)	
**Professional title**			0.069		0.009		0.482		0.191		0.294
Primary	1034 (62.0)	704 (68.1)		541 (52.3)		420 (40.6)		333 (32.2)		273 (26.4)	
Junior	511 (30.6)	354 (69.3)		262 (51.3)		195 (38.2)		179 (35.0)		118 (23.1)	
Senior	124 (7.4)	97 (78.2)		82 (66.1)		45 (36.3)		49 (39.5)		28 (22.6)	
**Department**			0.009		<0.001		0.891		0.457		0.533
Internal	485 (29.1)	345 (71.1)		277 (57.1)		192 (39.6)		161 (33.2)		119 (24.5)	
Surgical	333 (20.0)	255 (76.6)		202 (60.7)		135 (40.5)		118 (35.4)		91 (27.3)	
Obstetrics and Gynecology	111 (6.7)	71 (64.0)		67 (60.4)		48 (43.2)		41 (36.9)		29 (26.1)	
Pediatrics	120 (7.2)	81 (67.5)		52 (43.3)		48 (40.0)		43 (35.8)		35 (29.2)	
Acute and Severe unit	147 (8.8)	93 (63.3)		72 (49.0)		59 (40.1)		45 (30.6)		36 (24.5)	
Oncology	37 (2.2)	27 (73.0)		18 (48.6)		17 (45.9)		14 (37.8)		7 (18.9)	
Preventive healthcare	108 (6.5)	63 (58.3)		50 (46.3)		36 (33.3)		25 (23.1)		20 (18.5)	
Outpatient	53 (3.2)	35 (66.0)		25 (47.2)		19 (35.8)		19 (35.8)		17 (32.1)	
Other (traditional Chinese medicine and rehabilitation)	275 (16.5)	185 (67.3)		122 (44.4)		106 (38.5)		95 (34.5)		65 (23.6)	
**Institutions**			0.008		0.236		0.096		0.057		0.180
Tertiary A	921 (55.2)	654 (71.0)		492 (53.4)		385 (41.8)		333 (36.2)		248 (26.9)	
Secondary	387 (23.2)	276 (71.3)		217 (56.1)		149 (38.5)		126 (32.6)		93 (24.0)	
Township health center	237 (14.2)	153 (64.6)		117 (49.4)		87 (36.7)		67 (28.3)		48 (20.3)	
Community health center	124 (7.4)	72 (58.1)		59 (47.6)		39 (31.5)		35 (28.2)		30 (24.2)	
**Training on tobacco cessation**			<0.001		<0.001		<0.001		<0.001		<0.001
No	978 (58.6)	594 (60.7)		413 (42.2)		242 (24.7)		171 (17.5)		106 (10.8)	
Yes	691 (41.4)	561 (81.2)		472 (68.3)		418 (60.5)		390 (56.4)		313 (45.3)	

*Nurses’ smoking, exposure to secondhand smoke, and the 5As in smoking cessation*

The current smoker nurses accounted for 1.1%, while 80.8% of nurses had family members or friends who smoked. A total of 76.4% of nurses were exposed to secondhand smoking in a week; 23.4% of the nurses’ families or friends suffered from smoking related diseases. Nurses’ smoking, smoking related diseases of their families or friends did not affect the 5As (p>0.05). Nurses without smoker families or smoker friends assessed and arranged follow-up more frequently than those with smoker families or smoker friends. Nurses who were exposed to secondhand smoking in a week asked and advised more than those who were not (p<0.05). Smoking status of nurses, families and friends suffering from smoking related diseases or not had no impact on 5As behavior (p>0.05). See details for the relationship between the smoking of nursing staff and exposure to secondhand smoking and the 5As in Supplementary file Table 1.

*Nurses’ knowledge of health hazards of smoking and the benefits of quitting, univariate analysis of the attitudes towards and 5As in the participation in tobacco cessation*

Most nurses (88.0%–95.3%) knew the harm of smoking on health, such as the occurrence of diabetes ulcers (89.3%), the deterioration of muscle strength (88.7%), male impotence and erectile dysfunction (88.7%); 89.0%–96.8% of nurses knew the benefits of quitting smoking, such as retrieving lifetime lost due to smoking (92.8%) and reduced incidence of coronary heart disease (93.2%).

In terms of attitudes towards participation in smoking cessation, 60.5% of nursing staff showed positive attitude towards various aspects of tobacco cessation. Most nurses (84.2%) reported non-smoker nurses as role models and the responsibility of medical staff to control smoking (77.3%). A significant number of nurses (87.1%) also thought smoking cessation was an important health education program, and 79.7% of nurses were prepared to attend smoking cessation training. However, only 61.7% were confident in helping smokers quit. Not many nurses (58.9%) believed they had the necessary knowledge and skills, and 59.6% knew the tobacco cessation regulations.

Regarding the knowledge of the harmful effects of tobacco and the benefits of smoking cessation, the participants were divided into a higher score group and a lower score group according to the average score, and differences in their use of 5As were found. The higher score group performed more 5As than the lower score group in corresponding knowledge and attitudes. The differences were statistically significant (p<0.05) (Table 2).

**Table 2:** The relationship between knowledge and attitudes towards tobacco cessation and the 5As behaviors among nurses: A survey in 2022 from Chongqing, China (N=1669)

*Variable*	*Total*	*Asking*		*Advising*		*Assessing*		*Assisting*		*Arranging*	
	*n (%)*	*n (%)*	*p*	*n (%)*	*p*	*n (%)*	*p*	*n (%)*	*p*	*n (%)*	*p*
**Knowledge score**			0.014		0.007		0.001		<0.001		<0.001
Lower group (0–14)	342 (20.5)	218 (63.7)		159 (46.5)		109 (31.9)		83 (24.3)		53 (15.5)	
Higher group (15–16)	1327 (79.5)	937 (70.6)		726 (54.7)		551 (41.5)		478 (36.0)		366 (27.6)	
**Attitude score**			<0.001		<0.001		<0.001		<0.001		<0.001
Lower group (0–7)	659 (39.5)	384 (58.3)		291 (44.2)		150 (22.8)		100 (15.2)		49 (7.4)	
Higher group (8–10)	1010 (60.5)	771 (76.3)		594 (58.8)		510 (50.5)		461 (45.6)		370 (36.6)	

*Nursing staff’s knowledge of smoking cessation resource and the effect on 5As approach*

The nursing staff’s knowledge of smoking cessation resources was unsatisfactory. A total of 60.7% of nurses knew the smoking cessation guidelines, while 46.1% knew about smoking cessation phone lines; 37.3% were familiar with smoking cessation Apps, 38.0% knew smoking control drugs, while 35.8% knew smoking cessation websites. The knowledge of smoking cessation resources affected the use of 5As, and the differences were statistically significant (Table 3).

**Table 3:** The relationship between knowledge of smoking control resources and 5As behaviors among nurses: A survey in 2022 from Chongqing, China (N=1669)

*Variable*	*Total*	*Asking*		*Advising*		*Assessing*		*Assisting*		*Arranging*	
	*n (%)*	*n (%)*	*p*	*n (%)*	*p*	*n (%)*	*p*	*n (%)*	*p*	*n (%)*	*p*
**Tobacco cessation guideline**			<0.001		<0.001		<0.001		<0.001		<0.001
Don’t know	656 (39.3)	358 (54.6)		254 (38.7)		119 (18.1)		77 (11.7)		39 (5.9)	
Know	1013 (60.7)	797 (78.7)		631 (62.3)		541 (53.4)		484 (47.8)		380 (37.5)	
**Tobacco control phone lines**			<0.001		<0.001		<0.001		<0.001		<0.001
Don’t know	900 (53.9)	538 (59.8)		381 (42.3)		216 (24.0)		140 (15.6)		70 (7.8)	
Know	769 (46.1)	617 (80.2)		504 (65.5)		444 (57.7)		421 (54.7)		349 (45.4)	
**Tobacco cessation Apps**			<0.001		<0.001		<0.001		<0.001		<0.001
Don’t know	1047 (62.7)	639 (61.0)		463 (44.2)		273 (26.1)		180 (17.2)		92 (8.8)	
Know	622 (37.3)	516 (83.0)		422 (67.8)		387 (62.2)		381 (61.3)		327 (52.6)	
**Tobacco cessation websites**			<0.001		<0.001		<0.001		<0.001		<0.001
Don’t know	1072 (64.2)	659 (61.5)		482 (45.0)		286 (26.7)		191 (17.8)		107 (10.0)	
Know	597 (35.8)	496 (83.1)		403 (67.5)		374 (62.6)		370 (62.0)		312 (52.3)	
**Tobacco cessation drugs**			<0.001		<0.001		<0.001		<0.001		<0.001
Don’t know	1035 (62.0)	637 (61.5)		466 (45.0)		272 (26.3)		175 (16.9)		99 (9.6)	
Know	634 (38.0)	518 (81.7)		419 (66.1)		388 (61.2)		386 (60.9)		320 (50.5)	

*Influence of external environmental factors on smoking cessation and the use of 5As*

The majority of nursing staff (84.1%) thought smokers’ motivation to quit was very important, and 81.1% regarded that the work load without spare time significantly impacted the support for smoking control. In all, 82.6% of nursing staff believed that the support of the working institution for smoking cessation was very important. The differences were statistically significant (p<0.05). Table 4 gives the external factors affecting nurses’ tobacco cessation participation.

**Table 4:** External factors determining the implementation of 5As behaviors of nurses: A survey in 2022 from Chongqing, China (N=1669)

*Variable*	*Total*	*Asking*		*Advising*		*Assessing*		*Assisting*		*Arranging*	
	*n (%)*	*n (%)*	*p*	*n (%)*	*p*	*n (%)*	*p*	*n (%)*	*p*	*n (%)*	*p*
**Motivation of smokers**			<0.001	<0.001		<0.001		<0.001		<0.001	
Unimportant	265 (15.9)	126 (47.5)		94 (35.5)		56 (21.1)		45 (17.0)		26 (9.8)	
Important	1404 (84.1)	1029 (73.3)		791 (56.3)		604 (43.0)		516 (36.8)		393 (28.0)	
**Available time**			<0.001		<0.001		0.022		0.044		0.027
Unimportant	316 (18.9)	178 (56.3)		131 (41.5)		107 (33.9)		91 (28.8)		64 (20.3)	
Important	1353 (81.1)	977 (72.2)		754 (55.7)		553 (40.9)		470 (34.7)		355 (26.2)	
**Institution support for smoking cessation**			<0.001		<0.001		<0.001		<0.001		0.001
Unimportant	290 (17.4)	153 (52.8)		124 (42.8)		79 (27.2)		71 (24.5)		50 (17.2)	
Important	1379 (82.6)	1002 (72.7)		761 (55.2)		581 (42.1)		490 (35.5)		369 (26.8)	

### Regression analysis of determinants of the smoking cessation 5As implementation by nursing staff

Taking the participation in each of the 5As as the dependent variable, and taking factors with statistical significance (p<0.05) in the univariate analysis as the independent variable, binary logistic regression analysis was conducted backward to determine the factors that impacted 5As behavior. The analysis revealed, from the demographic characteristics, those who were more willing to ask the clients about smoking cessation: the females more than males (AOR=2.02; 95% CI: 1.01–4.05), those aged 40–49 years more than those aged 20–29 years (AOR=1.66; 95% CI: 1.08–2.56), and the married more than the divorced (AOR=2.23; 95% CI: 1.23–4.03). The higher ranked in professional titles were more willing to advise smokers to quit than the lower ranked (AOR=1.95; 95% CI: 1.28–2.99). A nurse with a Bachelor’s degree or higher delivered more assistance for smoking cessation to smokers (AOR=1.39; 95% CI: 1.05–1.83). The differences of working units were related to asking and advising. Compared to nursing staff in the internal medicine department, nurses in the preventive healthcare department (AOR=0.53; 95% CI: 0.33–0.85) rarely asked about the smoking status of clients, and those in pediatric (AOR=0.55; 95% CI: 0.35–0.84) and traditional Chinese medicine and rehabilitation departments (AOR=0.56; 95% CI: 0.40–0.77) rarely advised smokers to quit smoking. Having attended training or not (AOR=1.60; 95% CI: 1.22–2.11) was associated with all 5As. Nurses who had been exposed to secondhand smoking in a week asked (AOR=1.73; 95% CI: 1.33–2.25) and advised (AOR=1.42; 95% CI: 1.11–1.81) more about smoking cessation.

In terms of smoking cessation knowledge and attitudes, knowledge was correlated with advising, and attitude was a determinant of the four behaviors: asking (AOR=1.58; 95% CI: 1.24–2.01), assessing (AOR=1.94; 95% CI: 1.51–2.48), assisting AOR=2.35; 95% CI: 1.77–3.11), and arranging follow-up (AOR=3.47; 95% CI: 2.43–4.94).

Regarding tobacco cessation resources, knowledge of the tobacco cessation guidelines (AOR=1.75; 95% CI: 1.32–2.32) and Apps (AOR=1.50; 95% CI: 1.09–2.06) were determinants of all 5As. Knowledge of smoking cessation drugs promoted assessing (AOR=1.45; 95% CI: 1.04–2.01), assisting (AOR=2.30; 95% CI: 1.64–3.23) and arranging follow-up (AOR=2.07; 95% CI: 1.42–3.01). The knowledge of smoking cessation websites and phone lines had no influence on the 5As of nursing staff.

Among the determinants of nurses’ participation in tobacco cessation, smokers’ motivation to quit smoking was related to all the 5As (AOR=2.12; 95% CI: 1.60–3.02). Sufficient working time (AOR=1.36; 95% CI: 1.01–1.84) was correlated with advising. The support of the working institution in smoking cessation was related to asking (AOR=1.61; 95% CI: 1.17–2.20). Table 5 shows the determinants of 5As of nursing staff in smoking cessation.

**Table 5:** Binary logistic regression analysis of 5As smoking control behaviors of nurses: A survey in 2022 from Chongqing, China (N=1669)

*Variable*	*Asking*		*Advising*		*Assessing*		*Assisting*		*Arranging*	
	*p*	*AOR (95% CI)*	*p*	*AOR (95% CI)*	*p*	*AOR (95% CI)*	*p*	*AOR (95% CI)*	*p*	*AOR (95% CI)*
**Gender**										
Male (Ref.)		1								
Female	0.047	2.02 (1.01–4.05)								
**Age** (years)										
20–29 (Ref.)		1								
30–39	0.548	0.92 (0.69–1.22)								
40–49	0.022	1.66 (1.08–2.56)								
>50	0.111	2.02 (0.85–4.78)								
**Education level**										
Junior and lower (Ref.)								1		
Undergraduate and higher							0.021	1.39 (1.05–1.83)		
**Marital status**										
Divorced (Ref.)		1								
Married	0.008	2.23 (1.23–4.03)								
Unmarried	0.069	1.84 (0.95–3.56)								
**Professional title**										
Primary (Ref.)				1						
Junior			0.787	0.97 (0.77–1.22)						
Senior			0.002	1.95 (1.28–2.99)						
**Secondhand smoking exposure** (1 week)										
No (Ref.)		1		1						
Yes	<0.001	1.73 (1.33–2.25)	0.005	1.42 (1.11–1.81)						
**Department**										
Internal (Ref.)		1		1						
Surgical	0.214	1.25 (0.88–1.76)	0.392	1.14 (0.84–1.54)						
Obstetrics	0.070	0.65 (0.40–1.04)	0.483	1.17 (0.75–1.83)						
Pediatrics	0.269	0.77 (0.48–1.23)	0.006	0.55 (0.35–0.84)						
Acute and Severe unit	0.323	0.80 (0.52–1.24)	0.144	0.75 (0.50–1.11)						
Oncology	0.593	1.24 (0.56–2.72)	0.563	0.81 (0.40–1.64)						
Preventive healthcare	0.008	0.53 (0.33–0.85)	0.105	0.69 (0.44–1.08)						
Outpatient	0.540	0.82 (0.43–1.56)	0.282	0.72 (0.40–1.31)						
Others (traditional Chinese medicine and rehabilitation)	0.134	0.77 (0.54–1.09)	<0.001	0.56 (0.40–0.77)						
**Tobacco cessation attitude**										
Lower score (0–7) (Ref.)		1				1		1		1
Higher score (8–9)	<0.001	1.58 (1.24–2.01)			<0.001	1.94 (1.51–2.48)	<0.001	2.35 (1.77–3.11)	<0.001	3.47 (2.43–4.94)
**Obtained training**										
No (Ref.)		1		1		1		1		1
Yes	0.001	1.60 (1.22–2.11)	<0.001	2.09 (1.65–2.65)	<0.001	2.36 (1.85–3.01)	<0.001	2.65 (2.04–3.44)	<0.001	2.62 (1.95–3.52)
**Tobacco cessation guidelines**										
Don’t know (Ref.)		1		1		1		1		1
Know	<0.001	1.75 (1.32–2.32)	0.001	1.54 (1.19–1.99)	<0.001	2.02 (1.51–2.71)	0.003	1.69 (1.20–2.38)	0.045	1.57 (1.01–2.44)
**Smoking control drugs**										
Don’t know (Ref.)						1		1		1
Know					0.028	1.45 (1.04–2.01)	<0.001	2.30 (1.64–3.23)	<0.001	2.07 (1.42–3.01)
**Tobacco cessation App**										
Don’t know (Ref.)		1		1		1		1		1
Know	0.013	1.50 (1.09–2.06)	0.003	1.50 (1.15–1.96)	0.039	1.43 (1.02–2.01)	0.001	1.86 (1.31–2.65)	<0.001	3.08 (2.08–4.55)
**Smokers’ motivation**										
Unimportant (Ref.)		1		1		1		1		1
Important	<0.001	2.20 (1.60–3.02)	0.001	1.75 (1.27–2.41)	<0.001	2.33 (1.66–3.28)	<0.001	2.32 (1.58–3.42)	<0.001	2.89 (1.79–4.65)
**Institution support**										
Unimportant (Ref.)		1								
Important	0.003	1.61 (1.17–2.20)								
**Available time**										
Unimportant (Ref.)	1			1						
Important			0.041	1.36 (1.01–1.84)						

AOR: adjusted odds ratio. Binary logistic regression AOR comparison of whether the nursing staff asked about the smoking status of clients or not. Selected p<0.05 through univariate analysis. *Asking*: according to gender, age, marriage, department, institution, obtained training, secondhand smoking exposure (1 week), external environmental factors, help in adjusting to external factors of smoking. *Advising*: according to marriage, professional title, department, institution, obtained training, secondhand smoking exposure (1 week), external environmental factors, help in adjusting to external factors of smoking. *Assessing*: according to gender, marriage, obtained training, family or friend smokers, external environmental factors, help in adjusting to external factors of smoking. *Assisting*: according to age, marriage, education level, obtained training, external environmental factors, help in adjusting to external factors of smoking. *Arranging*: according to family or friend smokers, obtained training, external environmental factors, help in adjusting to external factors of smoking. *External environmental factors*: available time, motivation of smokers, and institution support.

## DISCUSSION

This study was the first survey about tobacco cessation support among nursing staff in Chongqing. Its results were higher than those reported in a Hong Kong study, which showed 59.1% for asking, 64.9% for advising, 35.1% for assessing, 13.5–38.9% for assisting, and 20.1% for arranging follow-up^12^. The difference might be explained by the period of Hong Kong study data collection which took place 10 years ago. In recent years, China publicly and strongly has advocated against tobacco use and administered a series of regulations aimed at tobacco cessation. Chongqing implemented a comprehensive public smoking ban in 2021, and nursing staff performed more smoking control interventions. However, ‘advising and assisting’ behaviors were less than found in Beijing and Hefei. For the frequency of engaging in 5As in Beijing and Hefei, Sarna et al.^11^ obtained 64.0% for asking, 85.1% for advising, 51.8% for assessing, 52.5% for assisting, and 17.26% for arranging follow-up. This might be because their investigation was conducted in tertiary teaching hospitals. In China, tertiary teaching hospitals are the finest and best-equipped hospitals in terms of both technology and staff, they are regarded as models of health promotion^15^. This study included nursing staff not only from tertiary teaching hospitals but also from community and township health centers, representing nursing staff levels in medical institutions at different levels in Chongqing. Therefore, it could be inferred that the current overall performance of 5As among the Chongqing nurses increased compared to earlier studies. However, the results were lower than those from a study in Malta^21^. Malta research found that 76.3% of medical professionals asked, 83.5% advised and 70.5% assessed, but only 40.9% assisted and 24.2% arranged follow-up. The difference may have resulted from the different occupations of the participants. In the Malta study, nurses accounted for only 48.1%, and doctors performed more smoking cessation 5As than nurses. Nevertheless, a study in Spain^22^ found that nurses were more involved in 5As behavior than doctors. Compared to before, the participation of Chongqing nursing staff in smoking control 5As behavior increased, but further improvement is needed to reach the level in other countries.

The knowledge and familiarity with tobacco cessation resources affected smoking cessation behaviors^23^. This study found that tobacco cessation resources greatly impacted nurses’ 5As, especially the knowledge of tobacco cessation guidelines. China had administered Clinical Smoking Cessation Guidelines and required all medical personnel to provide smoking cessation assistance to smokers. Nurses with good knowledge of those guidelines engaged in 5As more frequently. Additionally, with the popularity of smartphones in China, mobile applications were most commonly used for health promotion in healthcare centers. Currently, there are many mobile applications for smoking cessation^24,25^ that have shown good outcomes^26,27^ and have been used by nurses as part of their 5As. In addition, withdrawal symptoms associated with quitting were an obstacle to smokers. Timely administration of smoking control drugs could alleviate the symptoms and help them successfully navigate the most difficult early period of smoking cessation^28^. Therefore, the knowledge of smoking control drugs was a determinant for three of the 5As, assessing, assisting and arranging follow-up services.

The knowledge about the harm from tobacco use and the benefits of quitting smoking were not related to 5As, and this was different from other studies^12^. It might be related to the selection of knowledge. Although this study selected 16 knowledge points based on relevant literature, there was a wealth of tobacco knowledge. What knowledge decided 5As behavior was an issue that needs to be further explored in future study. This study showed that attitudes towards smoking cessation affected four of the 5As – asking, assessing, assisting, and arranging follow-up – consistent with previous studies^29^. However, in this study, Chongqing nurses showed less enthusiasm for participating in tobacco cessation than those in previous surveys^10^, and only 60.5% had positive attitudes towards tobacco cessation. As noted above, for 87.1% of nurses, tobacco cessation was an important part of health education, 77.3% saw it as their responsibility, 61.7% were confident in assisting smokers to quit, and 58.9% thought they had the knowledge and skills to help smokers quit. Although Chongqing implemented a public smoking ban in 2021, only 59.6% of Chongqing nurses were familiar with the smoking cessation regulations, lower than that from other similar studies in China^11,12^. Therefore, it is necessary to provide more tobacco cessation training for nurses, to improve their knowledge and skills and promote positive attitudes towards participation in smoking cessation 5As. This study indicates that only 41.4% of the nursing staff had attended smoking cessation training, and their use of the 5As approach was significantly higher than those who did not. Studies, therefore, suggested providing tobacco cessation education for nursing staff and students^10-14^. Presently, tobacco cessation education is part of compulsory courses for nursing students^30,31^. The 5As behavior change model has been integrated into the new undergraduate nursing textbooks^28^, and regular training on tobacco cessation skills would be provided. On-the-job nursing staff should be provided with up-to-date tobacco cessation information in order to improve their knowledge and ability, enhancing their confidence in participating in tobacco cessation, and increasing their use of the 5As approach for tobacco cessation. Consequently, more smokers could obtain support to quit.

This study also found that working units were determinants for two of the 5As: asking and advising. Compared with nurses in internal medicine, preventive healthcare departments delivered services mainly to healthy individuals, so fewer asked about smoking. Pediatric departments mainly care for children with few smokers. Therefore, advising about smoking cessation was rare. Traditional Chinese medicine and rehabilitation departments mainly cared for chronic disease patients, and these patients had better selfcare awareness and less smoking behavior during long-term disease recovery. So those nursing staff performed fewer smoking cessation behaviors. In China, most females are non-smokers, and the nursing staff is predominantly female, so the females are more likely to be asking the smoking status of clients. This result was consistent with other studies^10-12^.

The motivation of smokers to quit smoking had impact on nurses’ 5As smoking cessation behavior. Institutional support for smoking cessation affected Ask. And the available working time had impact only on Advising. This demonstrated that in a supportive environment, nurses were willing to provide smoking cessation assistance to those who needed it, regardless of whether they had time or not. Thus, in ensuring smoke-free hospitals in China, various regulations and tools were employed to encourage smoking cessation. All medical institutions set up smoke-free environments to provide more smoking cessation support for both nursing staff and patients, and impelled more nurses to help smokers to quit smoking.

Finally, it is difficult to draw any specific conclusions regarding nurses’ tobacco use. Perhaps the reason is that 98.9% of nurses in Chongqing do not smoke. Nurses with higher exposure to secondhand smoking more frequently asked about smoking status and advised smoking cessation, which indicated they were highly sensitive to secondhand smoking and hoped more people would stay away from smoking.

### Strengths and limitations

This study is the first survey of nursing staff’s smoking cessation support in Chongqing. After the implementation of the public smoking ban in Chongqing, some smokers continued smoking at home. In recent years, people spent more time at home because of the COVID-19 pandemic. Given the harm caused by secondhand smoking, the need for an at-home intervention increased. More people were willing to quit smoking, and the overall demand for smoking cessation increased^17^. There are more than 100 thousand nurses in Chongqing, a significant human resource for tobacco cessation support. This study assessed the correlation between the frequency of implementing 5As and the nursing staff’s basic characteristics, knowledge, resources, and attitude towards tobacco cessation This study provided reference for smoking cessation education of nursing staff, improving the related knowledge and skills of smoking cessation and enabling nurses to help more smokers quit.

This study has some limitations. First, relevant information was self-reported by nurses. Because of social expectations, it may contain false reports. Second, the cluster effect of hospitals was not taken into consideration. The survey did not include hospital names because it was anonymously conducted. This was to protect the nurses’ privacy and dispel their reservations from completing the questionnaire. Third, this study only selected samples in Chongqing. Although the participants were collected from different regions, different healthcare units, and different levels of hospitals, they only represent nurses in Chongqing not nursing staff in other regions of China and other countries. Finally, because this study is a cross-sectional survey, a causal inference could not be made.

## CONCLUSIONS

Nursing staff from various medical institutions engaged in different aspects of the tobacco cessation 5As. Knowledge and availability of tobacco cessation resources significantly affected the nursing staff’s participation in smoking cessation 5As support. Smoking cessation education should be provided to nursing staff following the tobacco cessation guidelines to improve their knowledge and skills and enhance their enthusiasm for promoting and participating in tobacco cessation support programs.

## Data Availability

The data supporting this research are available from the authors on reasonable request.
