# Supplementary material for: Study on the participation of nursing staff in tobacco cessation support and related influencing factors: A survey from Chongqing, China
Source: Tob Induc Dis. 2023 Oct 12;21:131. doi: 10.18332/tid/170753 (PMC10568683; doi:10.18332/tid/170753)
Supplement: Supplementary file 1 [file TID-21-131-s1.pdf]

Study on the Participation of Nursing Staff in Tobacco Cessation Support and Related Influencing Factors: A Survey from Chongqing, China

Supplementary Table 1. The relationship between smoking or exposure to second-hand smoking and the 5A behaviors among nurses: a survey in 2022 from Chongqing, China (N=1669)

Variable	Total	Asking		Advising		Assessing		Assisting		Arranging	
	N（%）	N（%）	P	N（%）	P	N（%）	P	N（%）	P	N（%）	P
Smoking			0.207		0.796		0.588		0.304		0.992
Current smoking	18（1.1）	10(55.6)		9(50.0)		6(33.3)		4(22.2)		4(22.2)	
NO smoking	1651（98.9）	1145(69.4)		876(53.1)		654(39.6)		557 (33.7)		415(25.1)	
Family smokers or friend smokers			0.673		0.922		0.015		0.085		0.002

Yes	1348 (80.8)	936(69.4)		714(53.0)		514(38.1)		440(32.6)		317(23.5)	
No	321(19.2）	219(68.2)		171(53.3)		146(45.5)		121(37.7)		102(31.8)	
exposed to secondhand smoke in a week			<0.001		0.016		0. 540		0.507		0.418
no	394(23.6)	244(61.9)		188(47.7)		161(40.9)		127(32.2)		105(26.6)	
Yes	1275(76.4)	911(71.5)		697(54.7)		499(39.1)		434(34.0)		314(24.6)	
Smoking related diseases of families or friends			0.522		0.889		0.148		0.533		0.513
Yes	390(23.4)	275(70.5)		208(53.3)		142(36.4)		126(32.3)		93(23.8)	
No	1279(76.6)	880(68.8)		677(52.9)		518(40.5)		435(34.0)		326(25.5)	

© 2023 Song J. et al.
